# A long-term survival case of adult undifferentiated embryonal sarcoma of liver

**DOI:** 10.1186/1477-7819-10-65

**Published:** 2012-04-27

**Authors:** Keita Noguchi, Hideki Yokoo, Kazuaki Nakanishi, Tatsuhiko Kakisaka, Yosuke Tsuruga, Hirofumi Kamachi, Michiaki Matsushita, Toshiya Kamiyama

**Affiliations:** 1 Department of Gastroenterological Surgery I, Hokkaido University Graduate School of Medicine, N15, W7, Kita-ku, Sapporo, Hokkaido, 060-8638, Japan

**Keywords:** Long-term survival, Adult undifferentiated embryonal sarcoma of liver, Curable

## Abstract

Undifferentiated embryonal sarcoma of the liver (USEL) is a rare malignant hepatic tumor with a poor prognosis that is usually observed in children (aged 6 to10 years) and rarely seen in adults. We present a case of USEL in a 27-year-old woman with no previous history of the disease. Laboratory tests performed on admission showed that the patient had mildly elevated levels of aspartate aminotransferase, alanine transaminase, alkaline phosphatase, lactate dehydrogenase, and γ-glutamyl transpeptidase. The levels of viral hepatitis and tumor serum markers were all within normal limits. Computed tomography showed a large mass involving the right lobe and the medial segment of the liver. Right trisectionectomy was performed. Microscopically, the tumor was composed of pleomorphic and polynuclear dyskaryotic cells in a myxoid stroma with focal eosinophilic globules and no clear differentiation to muscle. Histological diagnosis showed undifferentiated embryonal sarcoma. Adjuvant therapy with cisplatin, vincristine, doxorubicin, cyclophosphamide, and actinomycin D was initiated. We administered a high dose of etoposide to extract the patient’s peripheral blood stem cells and performed radiation therapy and peripheral blood stem cell transplantation. At 5-year follow-up, the patient was alive without any evidence of recurrence. Here, we describe the clinical and histopathological features of USEL as well as the therapeutic options for USEL in adults with this disease.

## Backgound

Undifferentiated embryonal sarcoma of the liver (UESL) is a rare and aggressive malignant tumor that is observed predominantly in children, with a peak incidence in the age group of 6 to 10 years [1]. UESL was differentiated from other sarcomas by Stocker and Ishak in 1978. Although UESL is generally seen in children, it can also occur in adults. Thus, regardless of the patient’s age, it is important to consider this entity in differential diagnosis of large hepatic masses. UESL is a potentially treatable tumor, and for this reason, timely pathological diagnosis and multidisciplinary therapy is crucial to increase the chances of long-term survival. However, the standard multidisciplinary therapy for UESL is a matter of debate. Therefore, cases that show long-term survival with multidisciplinary therapy have great clinical significance. In this report, we present our experience in treating a 27-year-old woman with UESL. The patient underwent complete resection of the tumor and postoperative multidisciplinary therapy, the details of which are reported below.

## Case Presentation

**Figure 1:**
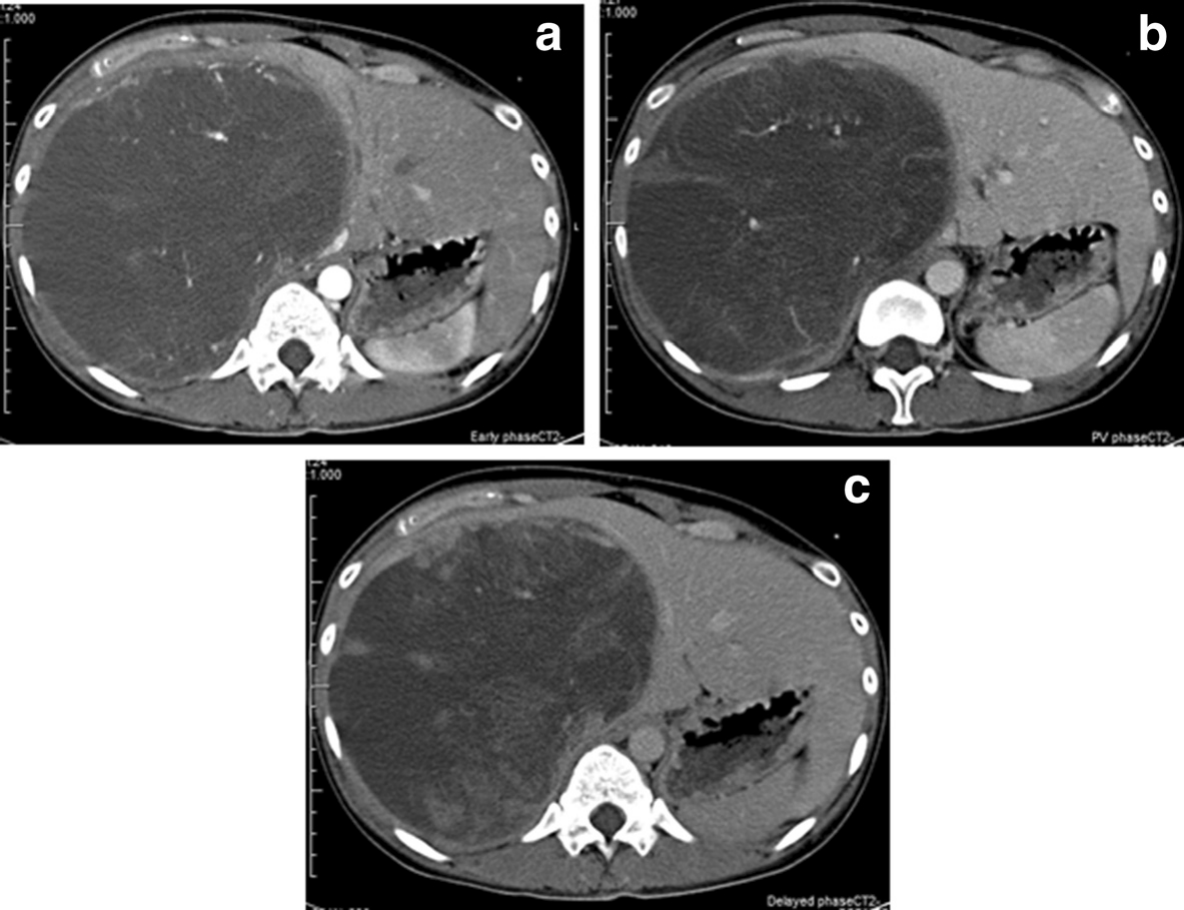
(a, b, and c) Abdominal computed tomography scan showing a well-defined, low-density large mass (21 × 19 × 14 cm) with heterogeneity in the right segments of the liver. This tumor had vessels, septa, no enhanced-effect, and no solid compartment (a, early phase; b, portal vein phase; and c, delayed phase).

**Figure 2:**
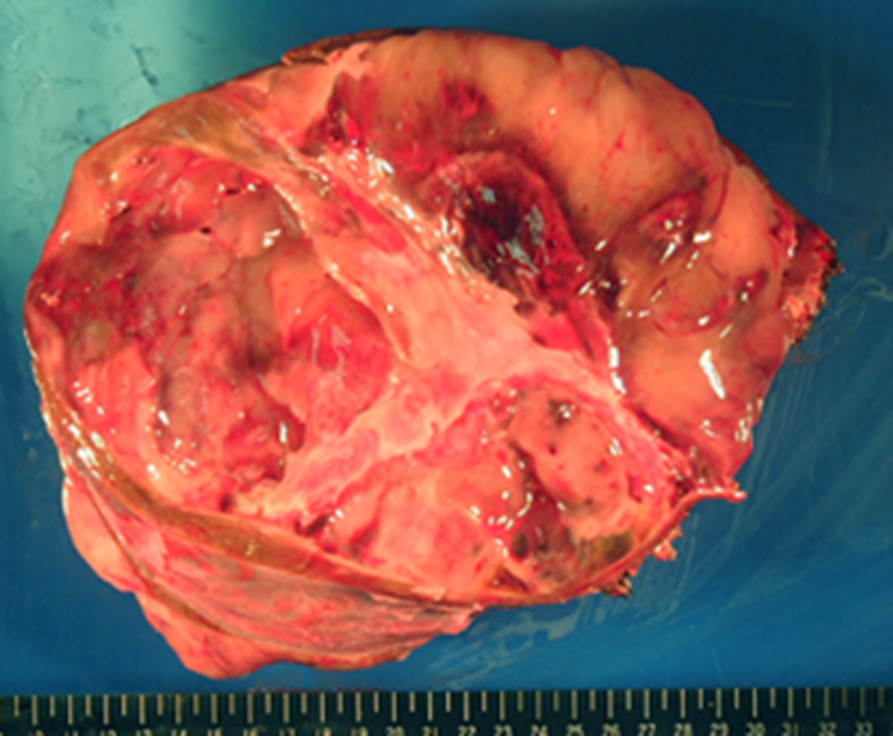
The cut surface showing a grayish-red soft mass with cystic degeneration, together with areas of hemorrhage and necrosis.

**Figure 3:**
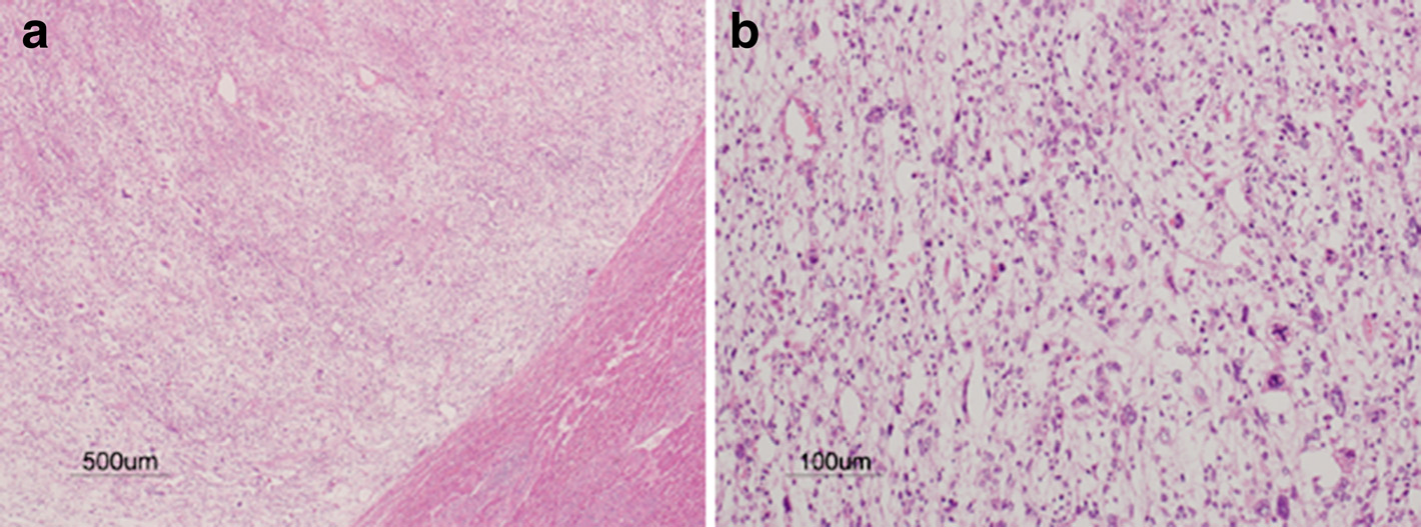
(a and b) The viscous area showing pleomorphic and polynuclear dyskaryotic cells in a myxoid stroma with focal eosinophilic globules (a, Hematoxylin-Eosin staining 4×, and b, 20×).

The patient was a 27-year-old Japanese woman who had right hypochondrial pain and low-grade fever. She consulted a local doctor when these symptoms aggravated. Computed tomography (CT) and ultrasonography showed a large hepatic tumor, and she was referred to our hospital for further examination and treatment. She was healthy and had no remarkable family history. Laboratory studies showed a normal serum bilirubin level and elevated levels of aspartate aminotransferase (AST) (42 U/L), alanine transaminase (ALT) (48 U/L), alkaline phosphatase (ALP) (660 U/L), lactate dehydrogenase (LDH) (398 U/L), and γ-glutamyl transpeptidase γ-(GPT) (204 U/L). Tests for serum viral hepatitis markers for hepatitis B and C yielded negative results. The levels of tumor markers, including α-fetoprotein (AFP), protein induced by vitamin K absence-II (PIVKA-II), carcinoembryonic antigen (CEA), carbohydrate antigen (CA) 19–9, and cancer antigen 125 (CA125), were within normal limits. Abdominal CT (Figure 1a, b, and c) revealed a well-defined low-density large heterogenous mass (21 × 19 × 14 cm) in the right liver segments. Enhanced CT revealed vessels, septa, and variable degrees of cystic change in the tumor and the absence of a solid compartment and enhanced-effect around the tumor. These findings indicated the possibility of UESL or mesenchymal hamartoma. Since her right hypochondrial pain worsened and the tumor enlarged rapidly, we considered that it was most likely UESL on the basis of the clinical symptoms, laboratory data, and CT findings. Considering the rapid progressive nature of UESL, right trisectionectomy was performed for both diagnosis and treatment on the day after admission. The tumor weighed 3,370 g. The cut surface revealed a grayish-red soft mass with cystic degeneration and areas of hemorrhage and necrosis (Figure 2). On microscopic examination, the tumor showed viscous, cystic, and grayish solid areas. The viscous area was composed of pleomorphic and polynuclear dyskaryotic cells in a myxoid stroma with focal eosinophilic globules (Figure 3a and b). The lumen in the cystic area had no epithelium. The grayish area was composed of myxoid stroma and atypical bile ducts and hepatic cord. Immunohistochemical analysis showed that the tumor did not express α-smooth-muscle actin (α-SMA), desmin, myoglobin, human hematopoietic progenitor cell marker (CD34), cytokeratin 19 antibody (AE-1/AE-3), hepatocyte paraffin 1 (HEP-PAR 1), and AFP, but it showed focal expression of Ki-67 (index 35 %) and mostly expressed p53. The differentiation to muscle was not clear in hematoxylin-eosin staining and other immunohistochemical analyses. The surgical margin was free. On the basis of these findings, a pathological diagnosis of UESL was made. After surgery, the patient received two courses of vincristine, adriamycin, cyclophosphamide, and actinomycin D (VADRCA) + cisplatin (CDDP) in accordance with the findings of The Third Intergroup Rhabdomyosarcoma study IRS-III [2] (Table 1). After the first course, the patient underwent a high-dose etoposide regimen for extraction of peripheral blood stem cells [3] (Table 2). During the second course, she received additional radiation therapy to remnant liver (Table 1). After the second course, she received ranimustine, carboplatin, etoposide, cyclophosphamide (MCVC) therapy and underwent peripheral blood stem cell transplantation [4] (Table 3). Follow-up imaging studies, including a whole-body CT scan and positron emission tomography (PET), showed no evidence of recurrence of the disease. She was healthy at the follow-up examination conducted 60 months after the surgical treatment.

**Table 1:** Adjuvant therapy: VADRCA + CDDP + RT

**Drug**	**(dose)**	**1**	**2**	**3**	**4**	**9**	**16**	**22**	**23**	**24**	**25**	**26**	**27**	**30**	**37**
CDDP	(90 mg/m^2^/day)	**↓**					**↓**								
VCR	(2 mg/m^2^/day)		**↓**			**↓**	**↓**		**↓**					**↓**	**↓**
ADM	(30 mg/kg/day)		**↓**	**↓**											
CTX	(10 mg/kg/day)		**↓**	**↓**	**↓**				**↓**	**↓**	**↓**				
ACT	(0.015 mg/kg/day)								**↓**	**↓**	**↓**	**↓**	**↓**		

Total course: two courses.

Radiation therapy with second course.

(20 Gray/10 fraction: day 6, 7, 9, 10, 13, 14, 16, 17, 20, 21).

ACT, actinomycin D; ADM, doxorubicin; CDDP, Cisplatin; CTX, cyclophosphamide; VCR, vincristine.

**Table 2:** Peripheral stem cell extraction

	Schedule			(day)
Peripheral stem cell extraction				**↓**
Drug (dose)	1	2	3	
VP-16 (500 mg/m^2^/day)	**↓**	**↓**	**↓**	

VP-16, etoposide.

**Table 3:** Peripheral stem cell transplantation

	Schedule (day)								
Peripheral stem cell transplantation									**↓**
Drug (dose)	−8	−7	−6	−5	−4	−3	−2	−1	
MCNU (200 mg/m^2^/day)	**↓**					**↓**			
CBDCA(300 mg/m^2^/day)		**↓**	**↓**	**↓**	**↓**				
VP-16 (500 mg/m^2^/day)			**↓**	**↓**	**↓**				
CTX (50 mg/kg/day)						**↓**	**↓**		

CBDCA, carboplatin; CTX, cyclophosphamide; MCNU, ranimustine; VP-16, etoposide.

## Discussion

The incidence of UESL is highest in children between 6 and 10 years of age [1]. To the best of our knowledge, in the past 50 years, less than 40 cases of UESL have been reported in patients older than 20 years [5]. UESL accounts for less than 1 % of all primary liver neoplasms in adults, and there are no specific clinical features and tumor makers. Furthermore, the results of imaging studies such as CT, ultrasonography, and magnetic resonance imaging (MRI) are often inconclusive. Thus, UESL must be considered during differential diagnosis of large liver tumors in children and adults. The diagnosis of UESL was delayed in some cases because the lesion presented as a large cystic hepatic mass, suggesting a benign lesion [6-12]. The prognosis of UESL is poor, but cases of long-term survival have been reported. Multidisciplinary treatment (chemotherapy and radiotherapy) has been used to achieve superior and local control and disease-free survival in patients with UESL [13,14]. For example, 17 children were reported to show long-term survival after UESL [14]. In this report, four patients who received complete resection followed by adjuvant chemotherapy achieved complete remission over a follow-up period of 5 years to 10.9 years. One patient was treated with vincristine, actinomycin, cyclophosphamide, and doxorubicin (VACA), two were treated with vincristine, actinomycin, ifosfamide, and doxorubicin (VAIA), and one was treated with ifosfamide, vincristin, and actinomycin (VAIA-IVA). In this report, it was noted that the response to chemotherapy was 62 %. In another report, three children who received complete resection followed by adjuvant chemotherapy of vincristine, actinomycin D, and cyclophsphamide (VADRC-VAC) + CDDP for 1 year, had a comparable prognosis with no evidence of the disease over a follow-up period of 40 to 60 months [15]. In another report, adjuvant chemotherapy after complete surgical resection of UESL showed significant therapeutic effects in adults [16]. In this report, all 14 patients who underwent complete tumor resection followed by adjuvant chemotherapy were alive after a median period of 28.5 months (range, 6–204 months). On the other hand, the 1-year and 2-year survival rates for all 14 patients who underwent complete tumor resection without receiving adjuvant chemotherapy was 53 %. Patients who underwent a complete tumor resection followed by adjuvant chemotherapy had significantly better survival rate than patients who underwent only surgery. In five patients who received incomplete surgical resection followed by chemotherapy, one patient was alive without any evidence of the disease after a follow-up of 6 months but all other patients died of sarcoma-associated complications after a median period of 12.5 months (range, 9–24 months). Three patients with UESL, who underwent incomplete surgical resection and received no postoperative chemotherapy, died after a median period of 12 months (range, 5–67 months). The difference in survival rates between these treatment groups was found to be statistically insignificant. Complete excision of the tumor seemed vital for cure. In addition, adjuvant chemotherapy after complete resection seemed be also important for cure. To our knowledge, only three adult cases of UESL (aged > 20 years) with patient survival for more than 60 months have been reported in literature [9,16,17] (Table 4). Complete resections were performed in two of these patients. In the third case, the UESL ruptured during the resection. After the operation, the cancer recurred and the patient underwent two more operations. However, he died because of recurrences at 67.6 months after the first liver resection [9]. In the other two cases of complete resection, postoperative chemotherapy was performed after complete resections. In one case, the patient had two lesions in the right and left liver. At first, she received hepatic right trisegmentectomy and was treated postoperatively with five cycles of intravenous ifosfamide (2,500 mg/m^2^, days 1–3), doxorubicin (50 mg/m^2^, day 2), and mensa (2,000 mg/m^2^, days 1–3). She also underwent resection of the left-side lesion and received two additional cycles of the chemotherapeutic regimen mentioned above. Both the resection margins were free. At 71 months after the second hepatic operation, she was healthy with no evidence of disease [17]. In the other case, the patient underwent an extended resection of the right liver lobe and partial resection of the diaphragm. The resection margin was free of tumor infiltration. She did not receive postoperative chemotherapy, and the tumor recurred 4 months later in the region of the former right liver lobe and the pelvis. She was treated with eight courses of intravenous carboplatin (at a dose of 150 mg/m^2^ on days 1–4) and etoposide (at a dose of 150 mg/m^2^ on days 1–4). After a gap of 4 weeks, she received chemotherapy with doxorubicin (at a dose of 30 mg/m^2^ on days 1–2) and ifosfamide (at a dose of 3,000 mg/m^2^ on days 1–3). The recurring lesions had reduced. Over a period of approximately 6 years, she received no further chemotherapy or surgical treatment and showed no evidence of recurrence [16]. No evidence of the disease was seen after complete resection and adjuvant chemotherapy in all three cases showing long-term survival; however, all the adjuvant chemotherapy regimens were different. Hence, we concluded that complete resection and adjuvant therapy were necessary for long-term survival, but the regimens of postoperative standard chemotherapy and radiation are a matter of debate. There are some reports that long-term survival cases of UESL with successful surgery, free margin and after conventional chemotherapy, but one case showed local recurrence despite successful surgery and adjuvant chemotherapy [16]. Although radiotherapy might be overtreated, we performed radiotherapy after obtaining the patient’s consent in this case. The findings of the cases of adult UESL, including this report, were vital for possible treatment options.

**Table 4:** Adjuvant therapy, recurrence and outcome of long-term survival cases of adult undifferentiated embronal sarcoma

**Reference**	**Year**	**Age**	**Resection margin**	**Adjuvant treatment**	**Recurrence**	**Treatment for recurrence**	**Follow-up months)**	**Outcome**
Grazi [9]	1996	25	Positive	None	Liver	Re-resection, two times	67.6	DOD
Alomogy [17]	2005	21	Negative	I + ADM, Re-resectionI + ADM	None		77	NED
Lenze [16]	2008	34	Negative	None	Liver	CBDCA + VP-16 + ADM + I	72	NED
Present	2011	27	Negative	DDP + VCR + ADM + ACTRT + PBSCT (VP-16, MCNU, CBDNU, CBDCA, CTX)	None		60	NED

ACT, actinomycin D; ADM, doxorubicin; CBDCA, carboplatin; CTX, cyclophosphamide; DDP, cisplatin; DOD, dead of disease; I, ifosfamide; MCNU, ranimustine, NED, no evidence of disease; PBSCT, pheripheral blood stem cell transplantation; RT, radiotherapy; VCR, vincritine; VP-16, Etoposide.

## Conclusion

In summary, it was important to achieve accurate and early diagnosis of UESL and include it in the differential diagnosis of large liver masses, regardless of the patient’s age. UESL is an aggressive tumor, but a combination of surgical, chemotherapeutic, and radiological treatment may prolong the patient’s survival. We believe that complete resection is a very important factor for ensuring long-term survival.

## Consent

Written informed conset was obtained from the patient for publication fo the Case report and any accompaning images. A copy of the written consent is available for review by the Editor in Chif of this journal.

## Competing interests

The author(s) declare that they have no competing interest.

## Authors’ contribution

H Yokoo and T Kamiyama was involved in the review of loterature, acquisition of data and drafting and completing the manuscript. K Nakanishi, T Kakisaka, Y Tsuruga, H Kamachi and M Matsushita participated in the critical review of the paper. All authors read and approved the final manuscript.

## Funding

This report did not receive any specific grant from any funding agency in the public, commercial, or not-for-profit sector.
